# Novel Immune Infiltrating Cell Signature Based on Cell Pair Algorithm Is a Prognostic Marker in Cancer

**DOI:** 10.3389/fimmu.2021.694490

**Published:** 2021-09-14

**Authors:** Hao Zhang, Zeyu Wang, Ziyu Dai, Wantao Wu, Hui Cao, Shuyu Li, Nan Zhang, Quan Cheng

**Affiliations:** ^1^Department of Neurosurgery, Xiangya Hospital, Central South University, Changsha, China; ^2^Department of Oncology, Xiangya Hospital, Central South University, Changsha, China; ^3^Department of Psychiatry, The Second People’s Hospital of Hunan Province, The Hospital of Hunan University of Chinese Medicine, Changsha, China; ^4^Department of Thyroid and Breast Surgery, Tongji Hospital, Tongji Medical College of Huazhong University of Science and Technology, Wuhan, China; ^5^One-third Lab, College of Bioinformatics Science and Technology, Harbin Medical University, Harbin, China; ^6^Department of Clinical Pharmacology, Xiangya Hospital, Central South University, Changsha, China; ^7^National Clinical Research Center for Geriatric Disorders, Xiangya Hospital, Central South University, Changsha, China

**Keywords:** immune cell, glioma microenvironment, cell pair algorithm, immunotherapy, prognostic model

## Abstract

Tumor-infiltrating immune cells (TIICs) have become an important source of markers for predicting the clinical outcomes of cancer patients. However, measurements of cellular heterogeneity vary due to the frequently updated reference genomes and gene annotations. In this study, we systematically collected and evaluated the infiltration pattern of 65 immune cells. We constructed the Immune Cell Pair (ICP) score based on the cell pair algorithm in 3,715 samples and across 12 independent cancer types, among which, the ICP score from six cancer types was further validated in 2,228 GEO samples. An extensive tumorigenic and immunogenomic analysis was subsequently conducted. As a result, the ICP score showed a robust reliability and efficacy in predicting the survival of patients with gliomas, in pan-cancer samples, and six independent cancer types. Notably, the ICP score was correlated with the genomic alteration features in gliomas. Moreover, the ICP score exhibited a remarkable association with multiple immunomodulators that could potentially mediate immune escape. Finally, the ICP score predicted immunotherapeutic responses with a high sensitivity, allowing a useful tool for predicting the overall survival and guiding immunotherapy for cancer patients.

## Introduction

Tumor-infiltrating immune cells (TIICs), including T cells, B cells, macrophages, and natural killer (NK) cells, represent the major components of immune response against a tumor (1). TIICs not only regulate the immunosurveillance and survival of cancer (2), but also accelerate tumor progression by creating a permissive microenvironment that stimulates tumor growth (3). Accumulating evidences have demonstrated that TIICs were associated with the clinical outcomes in various cancer types, including breast cancer (4), ovarian cancer (5), pancreatic tumor (6), lung adenocarcinoma (7), head and neck squamous cell carcinoma (8), and melanoma (9). However, efforts are still needed for a deep understanding of the immune activity of TIICs in the tumor microenvironment. So far, classic methods estimating TIICs include flow cytometry, immunofluorescence, and RNAseq. However, unified results may appear due to the different intervention factors in each method or different reference genomes. It should be noted that the fraction of each TIICs is within a relatively stable range. Thus, investigating the ratio of different TIICs is interesting and promising in optimizing the research about tumor microenvironment.

Previous studies have elucidated the tumor microenvironment in different cancer types, among which, glioma is one of the most common and malignant brain tumor with leading cancer-caused death rates. Currently, the clinical outcome of glioma patients is still dismal (10). Notably, glioma patients with similar clinical features tend to have a different prognosis due to the high level of heterogeneity, which greatly sets back the prospect for the prognosis of glioma patients. Previous studies have successfully extracted the TIICs from gliomas, aiming to provide a convincing evidence of the existence of abundant TIICs in gliomas microenvironment and provide important insights into immunotherapeutic approaches (11). The abundant available datasets of gliomas also facilitate the investigation on gliomas. Altogether, developing a TIIC-based signature in glioma and some other malignant cancer types can help in determining the prognostic value of TIICs, furthermore, improve the efficiency of immunotherapeutic approaches that activate the tumor-specific immune response.

In this study, 65 immune cell types were incorporated into the construction of the prognostic signature, the abundance of which was estimated in the glioma cohort and six independent cancer types to identify the immune cell types with an optimal prognostic value. Then, the immune cell pair (ICP) score was constructed based on the infiltration level of the identified results. ICP score was found to significantly correlate with the overall survival in glioma patients, six independent cancer types, and pan-cancer samples. Moreover, the ICP score profoundly correlates with various tumorigenic mutations in glioma patients and could sensitively predict the response to immunotherapy targeting immune checkpoints. This novel immune scoring system enables an in-depth understanding of tumor infiltrating immune cells and improves the clinical management of glioma patients.

## Materials And Methods

### Datasets Collecting and Preprocessing

Pan-cancer data and the corresponding clinical datasets were collected from the Gene Expression Omnibus (GEO; https://www.ncbi.nlm.nih.gov/geo/) and The Cancer Genome Atlas (TCGA; https://xenabrowser.net/). The glioma gene expression profiles and the corresponding clinical datasets were collected from the GEO, TCGA, and Chinese Glioma Genome Atlas (CGGA; http://www.cgga.org.cn/). A total of 5,230 pan-cancer samples of 12 independent cancer types were included in this study. A total of 3,715 glioma patient samples were collected from 14 cohorts. Cohorts with more than 50 glioma samples were included and cohorts with incomplete information on the overall survival of patients were excluded. In total, 2,228 samples of 12 cohorts consisting of 6 independent cancer types were from the GEO. The information of the platforms and numbers of samples of each cohort is provided in **Table S1**.

Raw data from the GEO datasets were generated using Affymetrix and Agilent. The robust multichip average (RMA) algorithm was used to perform quantile normalization and background correction of the raw data from Affymetrix. The consensus median polish algorithm was used for the final summarizing of oligonucleotides for each transcript in the Affymetrix software. Limma software was used for processing the raw data from Agilent. RNA-sequencing data were downloaded from the TCGA and CGGA data portals, and the fragments per kilobase million (FPKM) values were transformed into transcripts per kilobase million (TPM) values that had a similar signal intensity with the RMA-standardized values from the GEO datasets (12). R package sva was used to remove the computational batch effect among each cohort. Each cohort was processed and normalized independently.

### Immune Cell Signature Collection

Immune cell signatures were collected from diverse publicly available resources through a manually extensive literature search (13–22). Literatures with the reference genome of immune cells over the last 15 years were screened out. A total of 65 immune cell signatures were finally integrated by combining the gene sets of the same immune cell type from different literatures and excluding non-immune and non-stromal cell types. These 65 immune and stromal cells included B cells, CD8 T cells, DCs, Macrophages, Neutrophils, Th1 cells, Th2 cells, Mast cells, NK cells, Erythrocytes, Melanocytes, Megakaryocytes, Fibroblasts, Astrocytes, Basophils, Monocytes, Endothelial cells, et al. (**Table S2**). Thus, this immune cell signature was considered to be reliable and comprehensive.

### Development of a Reliable Prognostic Signature in Glioma

A prognostic signature was constructed based on stable immune infiltrating cells. The R package GSVA was applied to implement the single sample gene set enrichment analysis (ssGSEA) for calculating the immune enrichment score of 65 immune cell signatures in three glioma datasets, TCGALGGGBM-RNAseq (656 samples), CGGA311 (311 samples), and GSE108474 (414 samples), respectively (23). Univariate Cox analysis was performed on the 65 immune cell signatures to select the overlapped prognosis-associated immune cell types whose expression was significantly associated with patient OS (P < 0.05) in these three glioma datasets. Prognosis-associated immune cell types (Ci) were paired with all immune infiltrating cell types (Cj). For a cell pair started with Ci, Ci and Cj, Score_ij = 1 (exp_Ci – exp_Cj > 0) and Score_ij = 0 (exp_Ci – exp_Cj < 0). C-index was adopted to estimate the performance of each Score_ij and find out the Score_ij with the statistically significant p-value and highest C-index (16). For each Ci, Score_ij was identified with the highest C-index. For the obtained cell pairs, cell pairs were sorted with the HR > 1 and duplicate cell pairs were removed. Then, the ICP score was calculated as the sum of these selected Score_ij:

ICP score = Σ Score_ij

ICP score was then validated in all included 14 glioma cohorts and the Xiangya cohort.

### Genomic Alterations in the Immune Cell Pair Score

Somatic mutations and somatic copy number alternations (CNAs) which corresponded to the glioma samples with RNA-seq data were downloaded from TCGA. GISTIC analysis was adopted to determine the genomic event enrichment. CNAs associated with the two ICP score groups and the threshold copy number at alteration peaks were obtained using GISTIC 2.0 analysis (https://gatk.broadinstitute.org). The R package TCGAbiolinks was used for downloading the somatic mutation data derived from the WES data acquired by Mutect2 (24). Somatic mutations including the single-nucleotide variant (SNV), single-nucleotide polymorphism (SNP), insertion (INS), and deletion (DEL) were analyzed and visualized using the R package maftools (25). Based on the ascending order of the p-value, 30 most differentially mutated genes were detected using Fisher’s exact test. CoMEt algorithm was used to detect the co-occurrence and mutually exclusive mutations.

### Prediction of the Immune Cell Pair Score in Immunotherapy Response

The IMvigor210 cohort, a urothelial carcinoma cohort treated with the anti‐PD‐L1 antibody atezolizumab, was included for the prediction of response to immunotherapy (26). The melanoma dataset (GSE78220) was also used to predict the response to anti-PD-1 (pembrolizumab or nivolumab) immunotherapy (27). Based on the Creative Commons 3.0 License, complete expression data and clinical data were downloaded from http://research-pub.Gene.com/IMvigor210CoreBiologies. Raw data were normalized using the DEseq2 R package, and the count value or FPKM normalized value were transformed into the TPM value. ICP score was then constructed independently in these two datasets.

### Development of a Reliable Prognostic Signature in Other Cancer Types

Subsequently, the prognostic signature was constructed independently based on stable immune infiltrating cells in 12 cancer types from the pan-cancer data in TCGA. Univariate Cox analysis was used to select the prognosis-associated immune cell types whose expression was significantly associated with patient OS in each of the 12 cancer types (P < 0.05), respectively. Prognosis-associated immune cell types (Ci) were paired with all immune infiltrating cell types (Cj). For a cell pair starting with Ci, Ci and Cj, Score_ij = 1 (exp_Ci – exp_Cj > 0) and Score_ij = 0 (exp_Ci – exp_Cj < 0). C-index was adopted to estimate the performance of each Score_ij and find out the Score_ij with the statistically significant p-value and highest C-index (16). For each Ci, Score_ij was identified with the highest C-index. For the obtained cell pairs, cell pairs were sorted with the HR > 1 and duplicate cell pairs were removed. Then, the ICP score was calculated as the sum of these selected Score_ij:

ICP score = Σ Score_ij

Twelve datasets of six representative cancer types were selected for further validation of the ICP score established in each cancer type.

### RNA Sequencing

RNAstore-fixed tumor tissues from 48 glioma patients were collected for sequencing. RNA was sheared followed by sequencing library preparation using the NEBNext Ultra RNA Library Prep Kit. The Phusion High-Fidelity RNA polymerase, the Index (X) Primer and the Universal PCR primers. After target region capture by biotin-labeled probes, the captured libraries were sequenced on an Illumina Hiseq platform to generate 125/150 bp paired-end reads. In-house perlscripts were used to process raw data (raw reads). Then, reads containing adapter and ploy-N, and low-quality reads were removed to obtain clean data (clean reads). Reference genome and gene model annotation files were obtained from the genome website. Index of the reference genome was built using Hisat2 v2.0.5 and paired-end clean reads were aligned to the reference genome. FeatureCounts v1.5.0-p3 was then used to count the read numbers mapped to each gene. TPM value of each gene was calculated on the basis of the gene length and reads count.

### Statistical Analysis

Kaplan-Meier curves with log-rank test were used to assess survival difference between groups. The univariate and multivariate Cox regression analyses were performed to detect the prognostic factors. Pearson correlation analyses were used to calculate correlation coefficients. The cutoff value of ICP scores was calculated using the R package survminer. Based on the dichotomized ICP scores, patients were grouped as with high or low ICP score in each data set. Data was visualized using the R package ggplot2. OncoPrint was used to delineate the mutation landscape of TCGA by the maftools R package (28). All survivorship curves were generated using R package survminer. All statistical analyses were conducted using R software. P < 0.05 was considered statistically significant.

## Result

### Construction of the Immune Cell Pair Score and Its Prognostic Value

**Figure 1:**
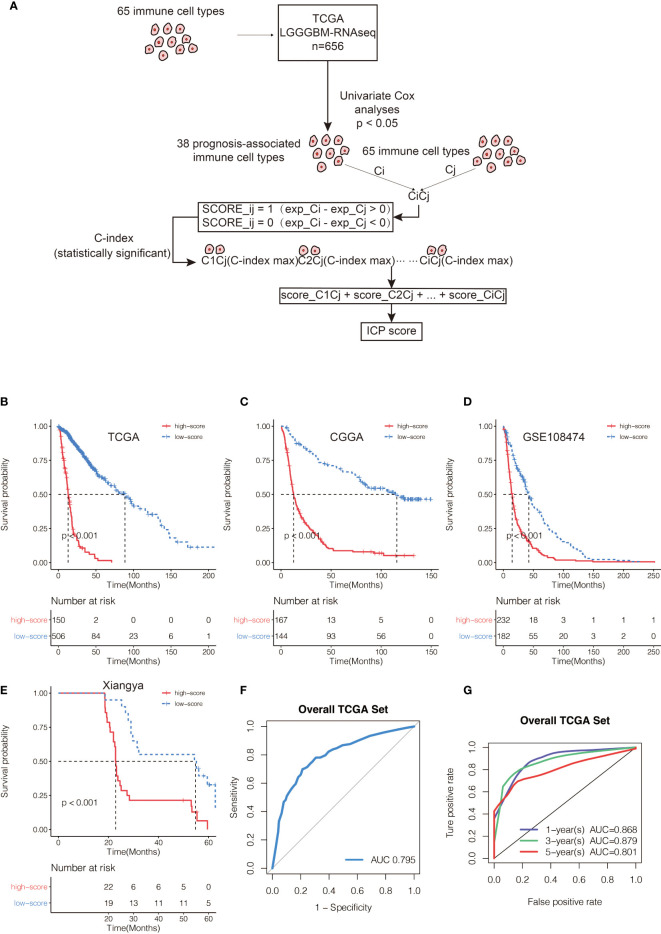
**(A)** Flow diagram of the cell pair algorithm. Kaplan–Meier curves for the two ICP score groups in **(B)** TCGA, **(C)** CGGA, and **(D)** GSE108474, respectively. Log-rank test, P < 0.001. **(E)** Kaplan–Meier curves for the two ICP score groups in the Xiangya cohort. Log-rank test, P < 0.001. **(F)** ROC curve measuring the sensitivity of ICP score in predicting the survival status of the patients. The area under the ROC curve was 0.795. **(G)** ROC curve measuring the sensitivity of ICP score in predicting the 1-year, 3-year, and 5-year survival of the patients. The area under the ROC curve was 0.868, 0.879, and 0.801, respectively.

**Figure 2:**
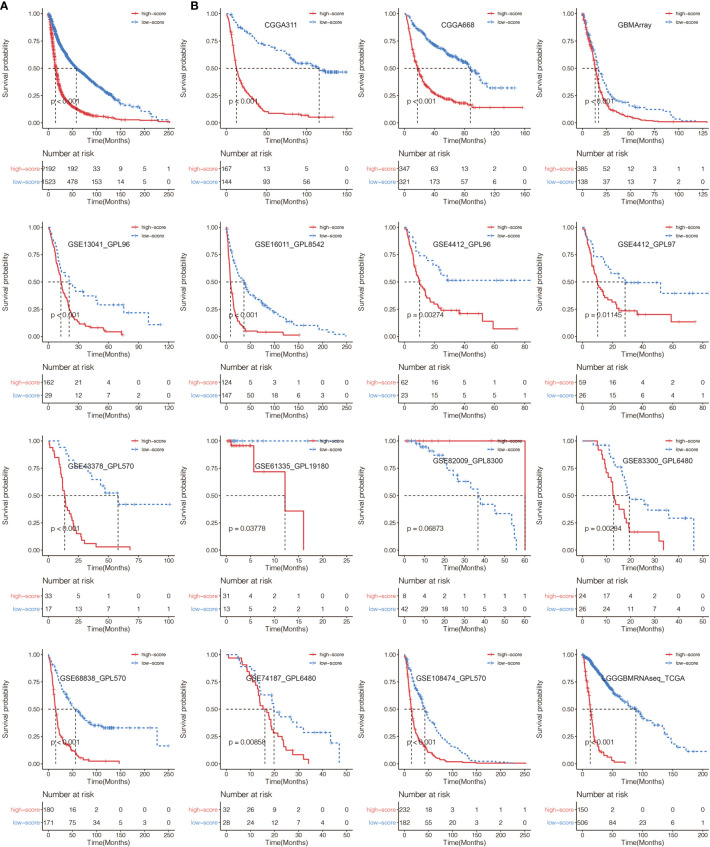
**(A)** Kaplan–Meier curves for the two ICP score groups in all glioma samples. Log-rank test, P < 0.001. **(B)** Kaplan–Meier curves for the two ICP score groups in all collected glioma datasets. Log-rank test, P < 0.05.

**Figure 3:**
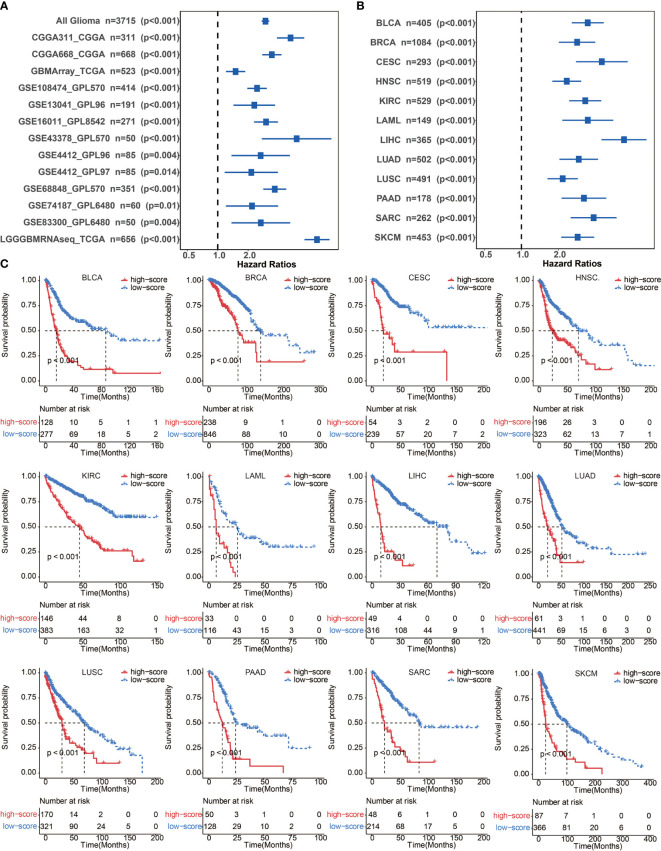
**(A)** Univariate cox regression analyses to estimate the clinical prognostic value between the low/high ICP score groups in independent glioma datasets. **(B)** Univariate cox regression analyses to estimate clinical prognostic value between low/high ICP score groups in 12 independent cancer types in TCGA. The length of the horizontal line represents a 95% confidence interval for each group. The vertical dotted line represents the hazard ratio (HR) in all patients. **(C)** ICP score was developed in 12 independent cancer types in TCGA. Kaplan–Meier curves for two ICP score groups in 12 cancer types. Log-rank test, P < 0.001. BLCA, Bladder Urothelial Carcinoma; BRCA, breast invasive carcinoma; CESC, Cervical squamous cell carcinoma and endocervical adenocarcinoma; HNSC, Head and Neck squamous cell carcinoma; KIRC, Kidney renal clear cell carcinoma; LAML, Acute Myeloid Leukemia; LIHC, Liver hepatocellular carcinoma; LUAD, Lung adenocarcinoma; LUSC, Lung squamous cell carcinoma; MESO, Mesothelioma; PAAD, Pancreatic adenocarcinoma; SARC, Sarcoma; SKCM, Skin Cutaneous Melanoma.

A total of 65 immune cell types were collected from publicly available resources and analyzed for the construction of ICP score. In total, 38 overlapped prognosis-associated immune cell types were identified by univariate Cox analysis performed on the 65 immune cell types in TCGA, CGGA, and GSE108474, respectively (**Table S3**). ICP score was calculated based on the predictive performance of each cell pair constituted from 38 prognosis-associated immune cell types and all 65 immune cell types (**Figure 1A**). Glioma patients were classified into high ICP score group and low ICP score group based on the cutoff value of the ICP scores calculated using the R package survminer. High ICP score was a prognostic marker for poor clinical outcomes in pan-glioma samples from TCGA, CGGA, and GSE108474 (log-rank test, p < 0.001; **Figures 1B–D**, respectively). High ICP score was also a prognostic marker for poor clinical outcomes in LGG, and GBM samples from TCGA (log-rank test, p < 0.001, p = 0.00195, respectively; **Figure S2A**), CGGA (log-rank test, p < 0.001, respectively; **Figure S2B**), and GSE108474 (log-rank test, p < 0.001, p = 0.05947; **Figure S2C**). Moreover, a high ICP score correlated with a worse survival probability in the Xiangya cohort (log-rank test, p < 0.001; **Figure 1E**, **Table S4**). The receiver operating characteristic (ROC) analyses with the Area Under the Curve (AUC) of 0.795 confirmed that ICP score was a prognostic biomarker in predicting the survival status of glioma patients (**Figure 1F**). Further, ICP score was a prognostic biomarker in predicting the 1-year, 3-year, and 5-year survival of glioma patients, which the AUC of ROC curve was 0.868, 0.879, and 0.801, respectively (**Figure 1G**). The prognostic value of ICP score was further verified in all 3,715 glioma samples included in this study (**Figure 2A**) and in each of the glioma datasets (**Figure 2B**). ICP score could significantly stratify the survival of glioma patients. The univariate Cox analyses confirmed that ICP score was a hazardous factor in glioma (**Figure 3A**).

### Validation of the Immune Cell Pair Score in Other Cancer Types

**Figure 4:**
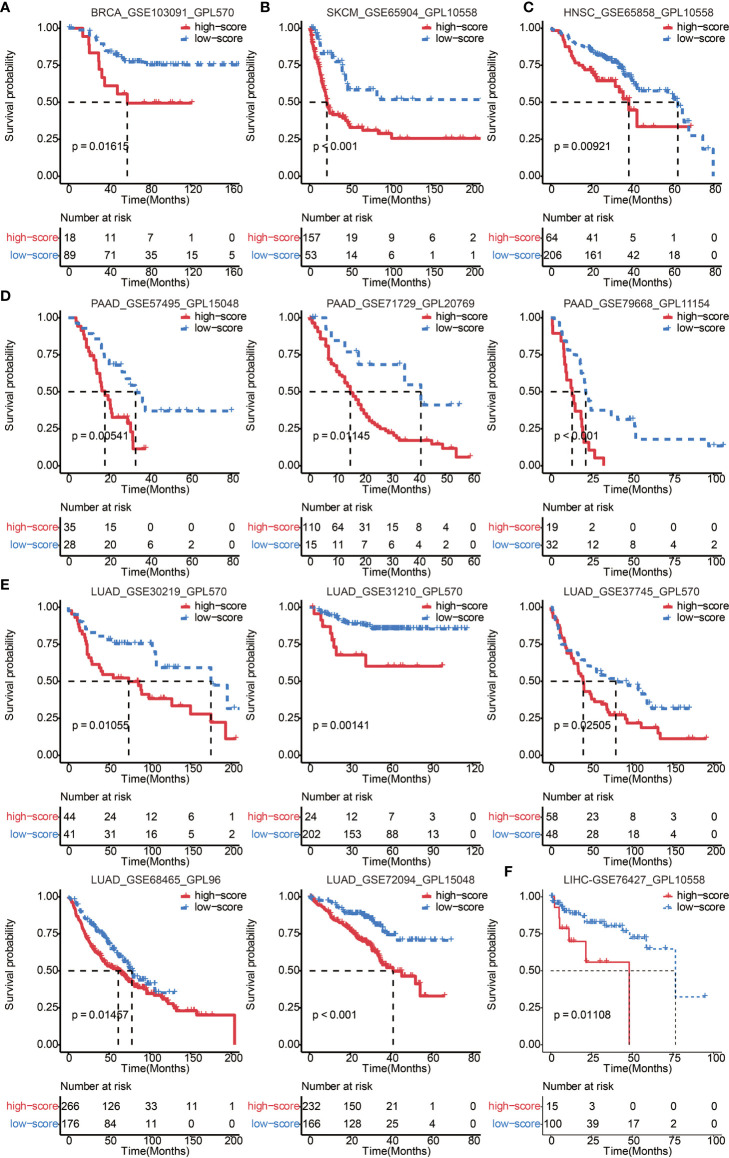
Validation of ICP score in 6 representative cancer types. **(A)** Kaplan–Meier curves for the two ICP score groups in the BRCA dataset, GSE103091. Log-rank test, P = 0.01615. **(B)** Kaplan–Meier curves for the two ICP score groups in the SKCM dataset, GSE65904. Log-rank test, P < 0.001. **(C)** Kaplan–Meier curves for the two ICP score groups in the HNSC dataset, GSE65858. Log-rank test, P = 0.00921. **(D)** Kaplan–Meier curves for the two ICP score groups in the PAAD datasets, GSE57495, GSE71729, and GSE79668. Log-rank test, P = 0.00541, P = 0.01145, and P < 0.001, respectively. **(E)** Kaplan–Meier curves for the two ICP score groups in the LUAD datasets, GSE30219, GSE31210, GSE37745, GSE68465, and GSE72094. Log-rank test, P = 0.01055, P = 0.00141, P = 0.02505, P = 0.01457, and P < 0.001, respectively. **(F)** Kaplan–Meier curves for the two ICP score groups in the LIHC dataset, GSE76427. Log-rank test, P = 0.01108.

To further confirm the efficacy and stability of the prognostic signature from the 65 immune cell types, ICP score was developed in 12 cancer types from TCGA, respectively. ICP score predicted a worse survival outcome in all of the 12 cancer types included (**Figure 3C**), and the univariate Cox analyses confirmed that ICP score was a hazardous factor in all of the 12 cancer types (**Figure 3B**). We then performed the validation of ICP score in six most representative cancer types (**Table S5**). As expected, ICP score was associated with a worse overall survival in breast cancer (**Figure 4A**), melanoma samples (**Figure 4B**), Head and Neck squamous cell carcinoma samples (**Figure 4C**), Pancreatic adenocarcinoma samples (**Figure 4D**), Lung adenocarcinoma samples (**Figure 4E**), and Liver hepatocellular carcinoma samples (**Figure 4F**).

### Genomic Features of the Immune Cell Pair Score Groups in Glioma

**Figure 5:**
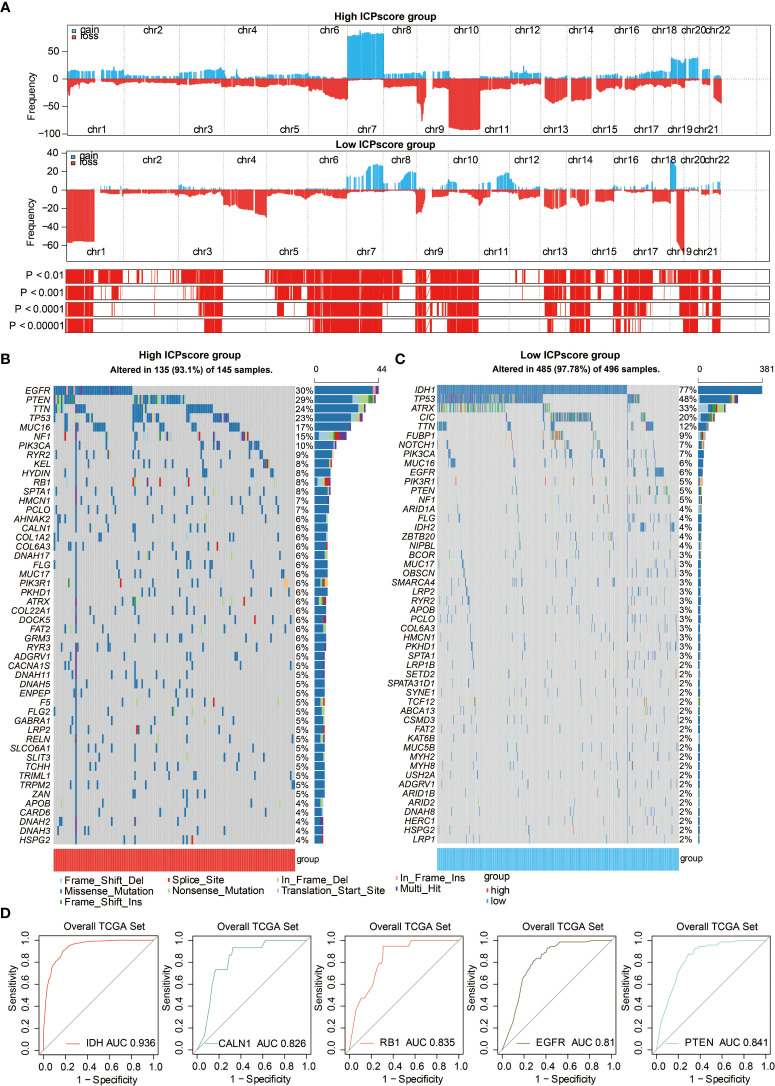
Genomic features of ICP score. **(A)** GISTIC 2.0 distribution of gain or loss of function mutation in gliomas with high and low ICP score. Chromosomal locations of peaks of significantly recurring focal amplifications (red) and deletions (blue) are presented. **(B)** List of the most frequently altered somatic mutation genes in the high ICP score group. **(C)** List of the most frequently altered somatic mutation genes in the low ICP score group. **(D)** ROC curve measuring the sensitivity of ICP score in predicting IDH, CALN1, RB1, EGFR, and PTEN mutation status. The area under the ROC curve was 0.936, 0.826, 0.835, 0.81, and 0.841, respectively.

Somatic mutation analysis and copy number variation (CNV) were performed using the TCGA dataset to explore the genomic traits of the two ICP score groups. A global CNV profile was obtained by comparing the two ICP score groups (**Figure 5A**, **Table S6**). According to somatic mutation analysis, mutations in EGFR (30%), TTN (24%), PTEN (29%), and TP53 (23%) were most highly enriched in the high ICP score group (**Figure 5B**). In comparison, IDH1 (77%), TP53 (48%), ARTX (33%), and CIC (20%) mutations were enriched in the low ICP score group (**Figure 5C**). Missense mutation was the predominant gene alteration type in all these genes except for ATRX, in which frame-shifting deletion was the most common type.

Different types of somatic mutations, including the single-nucleotide variant (SNV), single-nucleotide polymorphism (SNP), insertion, deletion, and intergenic region (IGR), were analyzed using the R package maftools. Silent, nonsense, missense, intronic, 5’ and 3’ UTR mutations were more common in the high ICP score group than in the low ICP score group (**Figure S3A**). While the frequencies of insertion and deletion were not statistically different between the two ICP score groups, SNPs were significantly more common in the high ICP score group (**Figure S3B**). Among the detected SNVs, C>T appeared to be the most common mutation in the high ICP score group (**Figure S3C**). The T to A, C to T,t and C to A mutations occurred more frequently in the high ICP score group than in the low ICP score group. The top 30 most differentially expressed mutated cancer-related genes between the two ICP groups are listed in **Figure S3D**. Common carcinogenic pathways were more active in the high ICP score group (**Figure S3E**). The strongest co-occurrent pairs of gene alteration in the high ICP score group were PTEN-TP53, RB1-TP53, TTN-CALN1, and TTN-FLG, which showed that TP53, PTEN, RB1, and TTN are functionally linked (**Figure S3F**). On the other hand, the most mutually exclusive pairs in the low ICP score group were CIC-TP53 and EGFR-IDH1 (**Figure S3F**). Furthermore, the AUC of ICP score for predicting the mutation status of IDH, CALN1, RB1, EGFR, and PTEN were 0.936, 0.826, 0.835, 0.81, and 0.841, respectively (**Figure 5D**).

### Potential Intrinsic Immune Escape Mechanisms Related to the Immune Cell Pair Score

**Figure 6:**
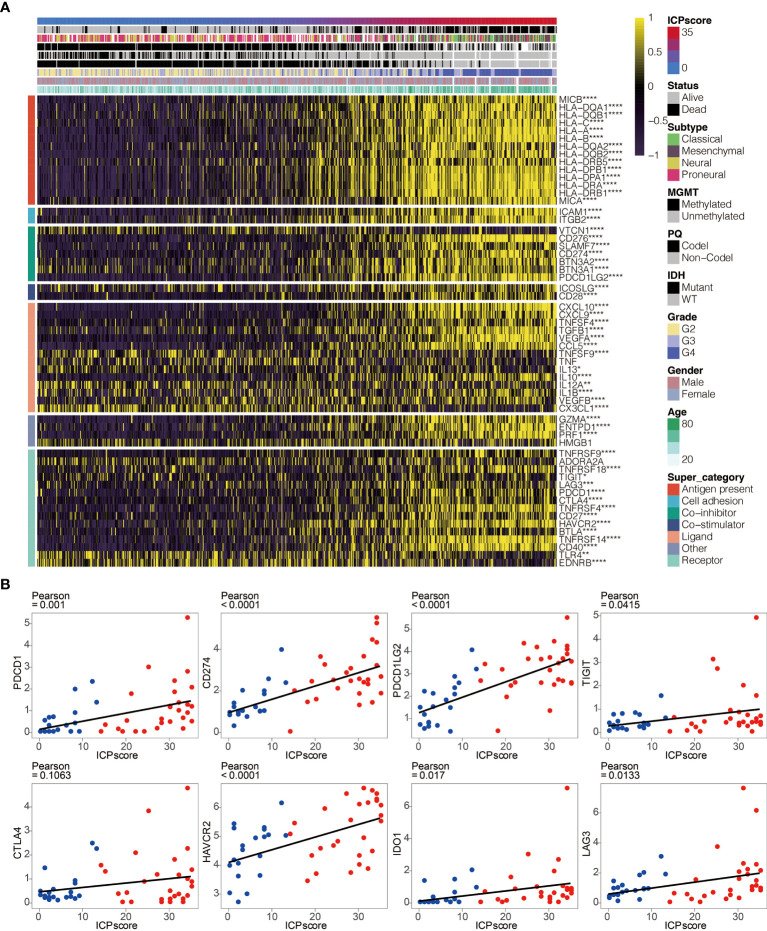
ICP score correlated with immune checkpoints. **(A)** Heatmap illustrating the expression pattern of immune checkpoints in ICP score. **(B)** Scatter plots depicting a positive correlation between ICP score and eight classical immune checkpoints, including PDCD1, CD274, PDCD1LG2, TIGIT, CTLA4, HAVCR2, IDO1, and LAG3. *P < 0.05; **P < 0.01; ***P < 0.001; ****P < 0.0001.

The intrinsic immune escape mechanism was reported to mainly include three aspects: immune checkpoint molecules, tumor immunogenicity, and antigen presentation capacity (29). We first explored the association between ICP score and immune checkpoint molecules which are classified into seven groups, including antigen-presenting, co-stimulator, co-inhibitor, and cell adhesion proteins and receptors, ligands, and others (3, 26). The increasing ICP score positively correlated with the expression of most immune checkpoint molecules (**Figure 6A**). In addition, ICP score had a significant positive relationship with some classical immune checkpoint molecules, including PDCD1, CD274, PDCD1LG2, TIGIT, HAVCR2, IDO1, and LAG3 in Xiangya cohort (**Figure 6B**).

**Figure 7:**
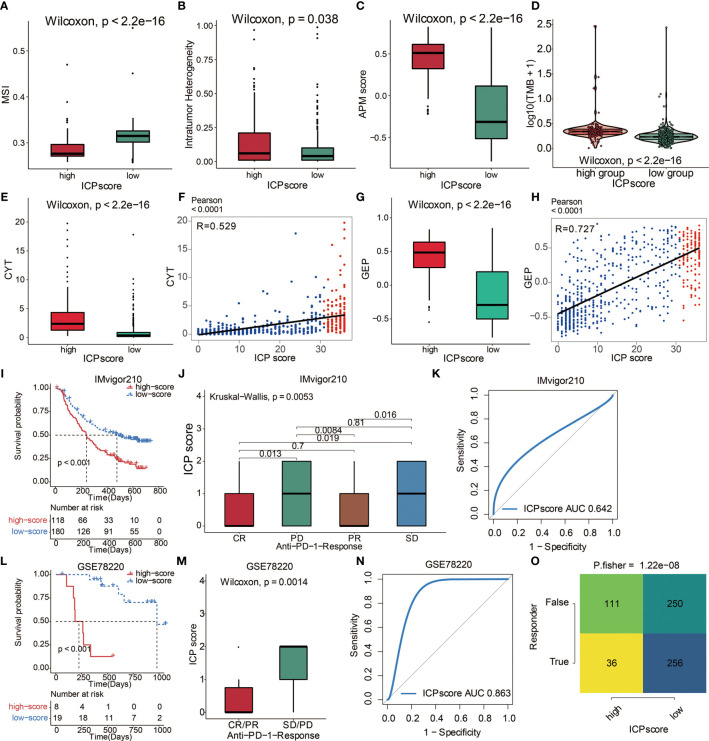
The predictive value of ICP score in immunotherapy. **(A)** MSI score in high and low ICP score. **(B)** APM score in high and low ICP score. **(C)** Intratumor Heterogeneity in high and low ICP score. **(D)** TMB expression differences in high and low ICP score. Differences between groups were compared through the Wilcoxon test (Wilcoxon, P < 0.001). **(E)** CYT and **(G)** GEP expression differences in high and low ICP score. Differences between groups were compared through the Wilcoxon test (Wilcoxon, P < 0.001). Scatter plots depicting a positive correlation between ICP score and **(F)** CYT and **(H)** GEP. Pearson Correlation Coefficient R = 0.529 and 0.727, respectively. **(I)** Kaplan–Meier curves for the two ICP score groups in the IMvigor210 dataset. Log-rank test, P < 0.001. **(J)** ICP score in groups with different anti–PD-1 clinical response status (CR, PR, SD, PD). Differences between groups were compared by Kruskal-Wallis test (Kruskal-Wallis, P = 0.0053). **(K)** ROC curve measuring the sensitivity of ICP score in predicting the survival status of patients in the IMvigor210 dataset. The area under the ROC curve was 0.642. **(L)** Kaplan–Meier curves for the two ICP score groups in the GSE78220 dataset. Log-rank test, P < 0.001. **(M)** ICP score in groups with different anti–PD-1 clinical response status (CR/PR and SD/PD). Differences between groups were compared by Wilcoxon test (Wilcoxon, P = 0.0014). **(N)** ROC curve measuring the sensitivity of ICP score in predicting the survival status of patients in the GSE78220 dataset. The area under the ROC curve was 0.863. **(O)** TIDE value and response to immunotherapy of patients with ICP score. Fisher’s test, P < 0.001.

A series of factors associated with tumor immunogenicity was then assessed (**Table S7**). The high ICP score group exhibited a lower microsatellite instability (MSI) and homologous recombination deficiency (HRD) (**Figure 7A**, **Figure S4A**). High ICP score group presented a higher level of intratumor heterogeneity, nonsilent mutation rate, number of segments, aneuploidy score, and fraction altered, all of which were significant indicators for genome alteration (**Figure 7B**, **Figures S4B–D**). Cancer testis antigen (CTA) and neoantigens were a vital source of tumor-specific antigens, and they were significantly different between the ICP score groups (**Figures S4F–H**). In term of antigen presentation capacity (**Table S7**), the high ICP score group presented a higher antigen processing and presenting machinery (APM) score and T cell receptor (TCR) (**Figure 7C**, **Figures S4I, J**). Stroma signatures including TGF-beta response, leukocyte fraction, CD8, interferon gamma (IFNG), interferon stimulated genes resistance signature (ISG.RS), and IFNG hallmark gene set (IFNG.GS) were higher in the high ICP score group (**Figures S4K–P**).

### Immune Cell Pair Score Predicted Immunotherapeutic Responses

Immunotherapy is innovating the treatment of several solid cancer types. The response rates of tumor to PD-1 inhibition are reported to be correlated with the TMB (30), Cytotoxic activity (CYT) (31), and T cell-inflamed gene expression profile (GEP) (32). To explore the predictive value of ICP score in immunotherapeutic response, we analyzed the correlation between ICP score and the above three immune markers. High ICP score group was found to have a higher TMB level (**Figure 7D**), CYT level (**Figure 7E**), and GEP level (**Figure 7G**). Furthermore, ICP score had a significantly positive correlation with CYT (**Figure 7F**) and GEP (**Figure 7H**). The ability of the ICP score to predict the response of patients to immune-checkpoint therapy was explored by assigning the IMvigor210 cohort patients (urothelial carcinoma dataset) to different ICP score groups (**Table S8**). Patients receiving atezolizumab as the anti‐PD‐L1 therapy with a high ICP score exhibited a significantly shorter OS compared to patients with a low ICP score (**Figure 7I**). Patients with a low ICP score exhibited better immunotherapeutic responses (**Figure 7J**). ICP score was a prognostic biomarker in predicting patient survival status in the IMvigor210 cohort (**Figure 7K**). In the melanoma dataset, GSE78220, patients receiving either pembrolizumab or nivolumab as the anti-PD-1 therapy with a high ICP score also exhibited a significantly shorter OS compared to patients with a low ICP score (**Figure 7L**; **Table S9**). Likewise, patients with a low ICP score exhibited better immunotherapeutic responses (**Figure 7M**). ICP score was also a prognostic biomarker in predicting patient survival status in the GSE78220 dataset (**Figure 7N**). Meanwhile, the TIDE analyses proved that a high ICP score was less sensitive to anti-PD1 therapy and anti-CTLA4 therapy (**Figure 7O**).

## Discussion

Tumor infiltrating immune cells have been critical in tumorigenesis by exerting the two-sided effect that both regulates the immunosurveillance of cancer and creates a favorable microenvironment for cancer cell survival. Previous studies have demonstrated the prognostic value of several TIICs in different cancer types (33, 34). However, the overall survival under the influence of TIICs in cancers have not been adequately determined and a consensus-oriented prognostic signature regarding TIICs has not been reached. Moreover, considering the differences in the reference genomes and gene signatures of immune cells used for quantifying RNA-sequencing data, multiple previous prognostic models may have limitations in the cross-validation of different transcriptional datasets or different cancer types. The measurements of cellular heterogeneity vary due to the frequent updated version of annotation for immune cells and reference genome, which may impede their extensive application and set back the prospect for clinical practice (**Figure S5**) (35, 36). To resolve this issue, we collected and integrated 65 immune cells to establish a robust and comprehensive prognostic signature with the concept of cell pair. As mentioned in the method section, we focused on the relative expression level of immune cells for the quantification of the ICP score, which extensively reduced the effect of the updated annotation of the reference genome, eliminated the need for data normalization, and increased the accuracy in designing the signature.

In this study, given the malignancy of gliomas and abundant publicly available datasets, ICP score was first established in glioma samples. ICP score could significantly stratify the overall survival of glioma patients from TCGA and CGGA. Based on the sequencing data from Xiangya, high ICP score was associated with a worse survival in glioma patients. Consistently, high ICP score predicted a worse survival in the other 15 external glioma datasets. The independent establishment of ICP score was performed in 12 representative cancer types including BLCA, BRCA, CESC, HNSC, KIRC, LAML, LIHC, LUAD, LUSC, MESO, PAAD, SARC, and SKCM, all of which proved the predictive value of ICP score. The univariate cox regression analysis proved that ICP score was a hazardous marker in both glioma samples and 12 independent cancer types. Furthermore, six most representative cancer types including BRCA, SKCM, HNSC, PAAD, LUAD, and LIHC were selected for the validation of the ICP score. As expected, ICP score served as a hazardous marker, and the predictive value of ICP score was stable in all of the 12 GEO datasets. The findings above proved the generality and reliability of ICP score in predicting the prognosis of cancer patients.

Furthermore, the genomic features of ICP score were annotated in gliomas. The present study finds that the IDH1 missense mutations are overrepresented in the low ICP score group (77%), in accordance with previous findings that IDH mutations are more enriched in low grade gliomas and confer better survival outcomes in glioma patients (37). Likewise, tumor suppressor TP53, inhibiting GBM malignancy (38), was found to be more frequently mutated in the low ICP score group (48%). Conversely, EGFR, which is the most enriched mutated gene in the high ICP score group (30%) and whose alteration occurs in less than 6% of the low ICP score group as identified by somatic mutation analysis, has been reported to be frequently activated in GBM and predict worse survival outcomes in glioma patients (39). Another critical oncogene, PTEN (33), also had higher mutation rates in the high ICP score group (29%), implying a more malignant feature of the high ICP score group. Commonly mutated cancer-related genes were found to be more frequently expressed in high ICP score group, with PTEN-TP53, RB1-TP53, TTN-CALN1, and TTN-FLG being the strongest co-occurrent pairs of gene alteration. PTEN (40), TP53, RB1 (41), CALN1 (42), EGFR (43), and TTN (44) have been previously reported to play a role in tumorogenesis, in which ICP score exhibited a high sensitivity in predicting the mutation status of IDH, CALN1, RB1, EGFR, and PTEN. Thus, ICP score may be a potential predictor for the oncogenic process.

The potential immune escape mechanisms of ICP score were summarized and underlined. Immune checkpoint blockage (ICB) therapy targeting immune checkpoint molecules have demonstrated remarkable benefits (45). The significant correlation between ICP score and classical immune checkpoint molecules such as PDCD1, CD274, TIGIT, and LAG3 suggested that ICP score could be an effective indicator for immune checkpoint blockage (ICB) therapy (46–49). Moreover, high ICP score prominently participated in the regulation of immunomodulators for tumor immunogenicity and antigen presentation capacity. Low MSI, a diagnostic phenotype with more malignancy of cancer (50), was more significantly correlated with a high ICP score. High ICP score was also detected with higher Intratumor Heterogeneity, a diagnostic phenotype with more malignancy of cancer (51). Additionally, a high ICP score had the distinct biological characteristics regarding stroma signatures such as TGF-beta response, leukocyte fraction, and ISG.RS compared with a low ICP score, and these stroma signatures have previously been proved to facilitate the immune escape of cancer (52). The findings above suggested a novel orientation for the inclusion of ICP score as the indicators of immunosuppression.

Immunotherapy, represented by ICB, has become increasingly promising in tumor treatment. Notably, the IMvigor210 cohort and the melanoma dataset (GSE78220) treated with the anti‐PD‐L1 antibody atezolizumab have demonstrated remarkable clinical outcomes (26, 27). ICP score was then validated in these two datasets regarding its predictive value of the response to immunotherapy. As expected, a high ICP score correlated with a worse survival in both cohorts and predicted a worse response to immunotherapy. Further, high ICP score correlated with higher levels of TMB, CYT, and GEP, all of which are valuable markers in predicting immunotherapeutic response. Taken together, our findings revealed the robust value of ICP score in predicting immunotherapy efficacy.

Of note, more comprehensive analysis of multi-omics analysis about the functional annotation of immune signature will greatly complement the findings in this study and ensure the prospective application of the ICP scoring system. To the best of our knowledge, we are the first one to collect the comprehensive immune cell types in cancer and introduce the concept of cell pair for the establishment of a robust immune signature. The relative stable ratio of TIICs regarding their abundance in tumor microenvironment ensures the extensive application and high sensitivity of this immune signature, and will undeniably help understand tumor microenvironment and TIICs effects on immunotherapy.

## Data Availability Statement

The original contributions presented in the study are included in the article/**Supplementary Material**. Further inquiries can be directed to the corresponding authors.

## Author Contributions

HZ, QC, NZ, and ZW designed and drafted the manuscript. HZ, QC, NZ, HC, SL, WW, and ZD wrote figure legends and revised the manuscript. QC, HZ, and NZ conducted the data analysis. All authors contributed to the article and approved the submitted version.

## Funding

Financial support was provided by the National Natural Science Foundation of China (NO. 82073893, 81703622), China Postdoctoral Science Foundation (NO. 2018M633002), Hunan Provincial Natural Science Foundation of China (NO. 2018JJ3838), and Hunan Provincial Health Committee Foundation of China (C2019186). Xiangya Hospital Central South University postdoctoral foundation. Fundamental Research Funds for the Central Universities of Central South University (2021zzts1027).

## Conflict of Interest

The authors declare that the research was conducted in the absence of any commercial or financial relationships that could be construed as a potential conflict of interest.

## Publisher’s Note

All claims expressed in this article are solely those of the authors and do not necessarily represent those of their affiliated organizations, or those of the publisher, the editors and the reviewers. Any product that may be evaluated in this article, or claim that may be made by its manufacturer, is not guaranteed or endorsed by the publisher.

## Abbreviations

TIICs, tumor-infiltrating immune cells; ICP, immune cell pair; NK, natural killer; GEO, Gene Expression Omnibus; TCGA, The Cancer Genome Atlas; CGGA, Chinese Glioma Genome Atlas; RMA, robust multichip average; FPKM, fragments per kilobase million; TPM, transcripts per kilobase million; SNV, single-nucleotide variant; SNP, single-nucleotide polymorphism; INS, insertion; DEL, deletion; ROC, receiver operating characteristic; AUC, area under the curve; CNV, copy number variation; MSI, microsatellite instability; HRD, homologous recombination deficiency; CTA, cancer testis antigen; APM, antigen processing and presenting machinery; TCR, T cell receptor; IFNG, interferon gamma; ISG.RS, interferon stimulated genes resistance signature; IFNG.GS, IFNG hallmark gene set; CYT, cytotoxic activity; GEP, T cell-inflamed gene expression profile; ICB, immune checkpoint blockage.
