# Supplementary material for: Novel Immune Infiltrating Cell Signature Based on Cell Pair Algorithm Is a Prognostic Marker in Cancer
Source: Front Immunol. 2021 Sep 14;12:694490. doi: 10.3389/fimmu.2021.694490 (PMC8476752; doi:10.3389/fimmu.2021.694490)
Supplement: Supplementary file 10 [file DataSheet_10.docx]

**Supplementary Figures**


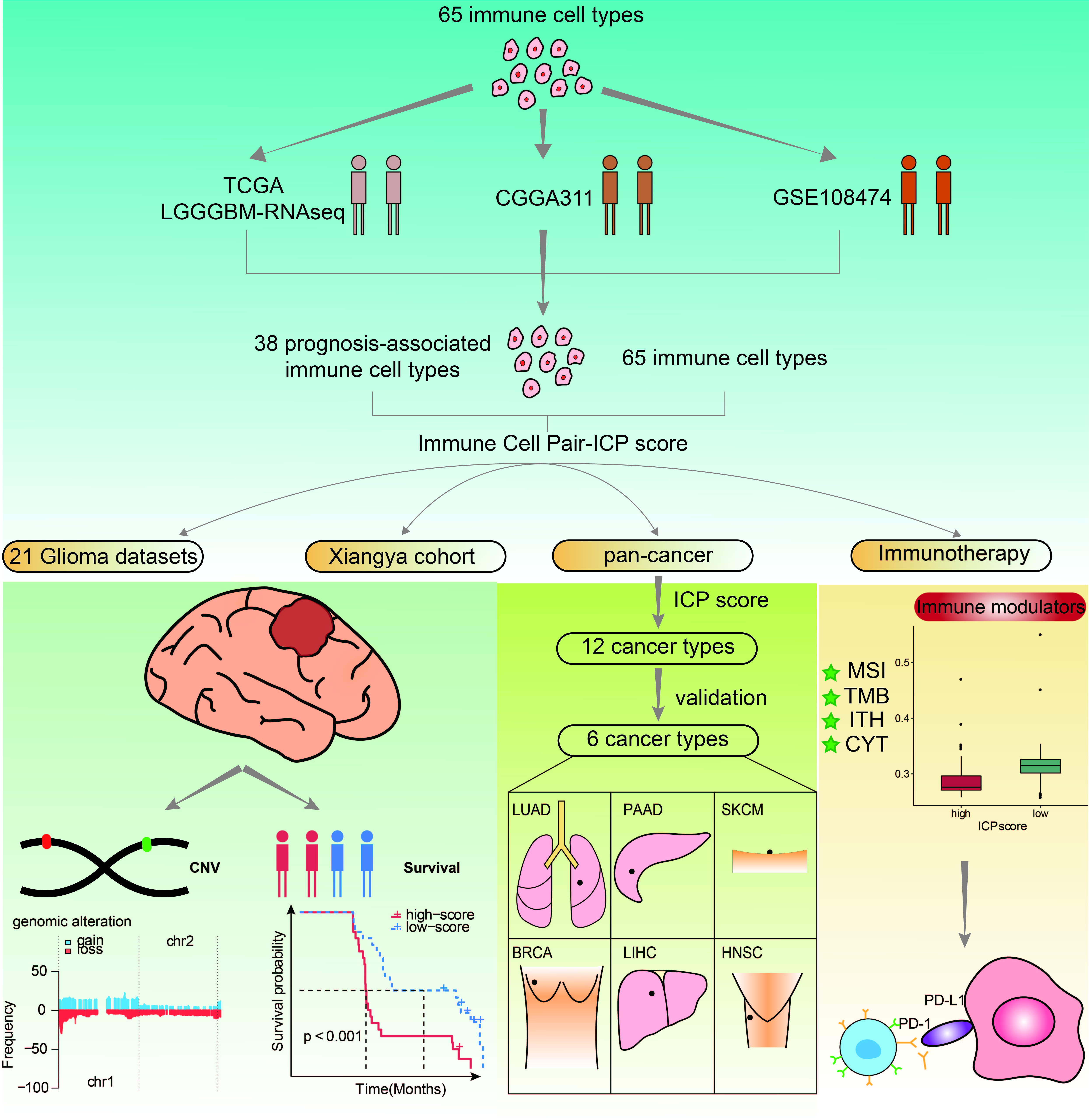


Figure S1. Flow diagram of this study. The infiltration pattern of 65 immune cell in 3715 glioma samples and 5603 TCGA pan-cancer samples were systematically evaluated. Immune cell pair (ICP) score was constructed based on the cell pair algorithm in three glioma cohort, and verified in glioma samples, Xiangya cohort, and TCGA pan-cancer samples. Immune cell pair (ICP) score was then constructed in six independent cancer types in TCGA and verified in 2228 GEO samples. An extensive tumorigenic and immunogenomic analysis were conducted. Immune cell pair (ICP) score predicted immunotherapy response.


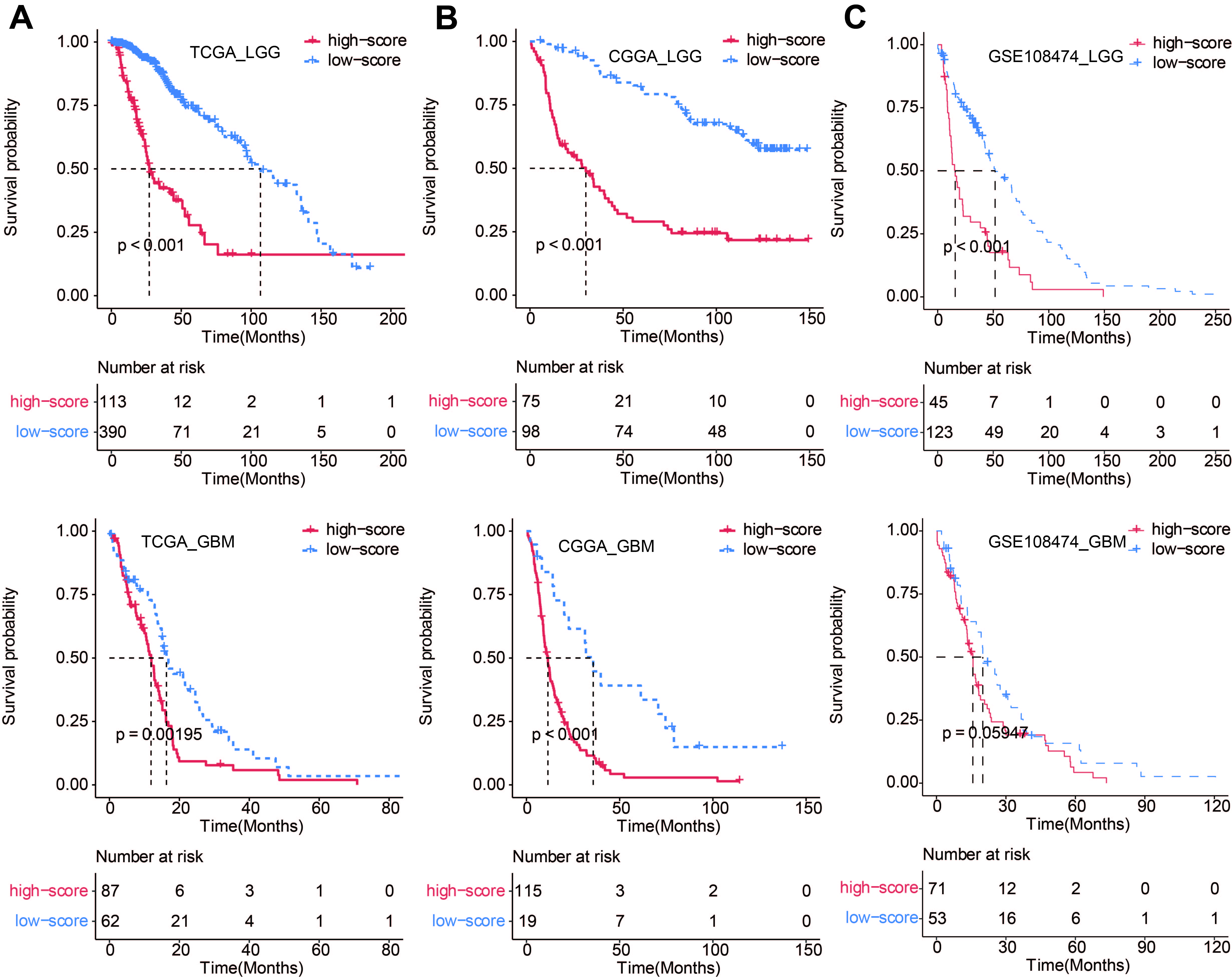


Figure S2. **A**. Kaplan–Meier curves for two ICP score groups in TCGA LGG, and GBM, respectively. Log-rank test, P < 0.001, P = 0.00195, respectively. **B**. Kaplan–Meier curves for two ICP score groups in CGGA LGG, and GBM, respectively. Log-rank test, P < 0.001, respectively. **C**. Kaplan–Meier curves for two ICP score groups in GSE108474 LGG, and GBM, respectively. Log-rank test, P < 0.001, P = 0.05947, respectively.


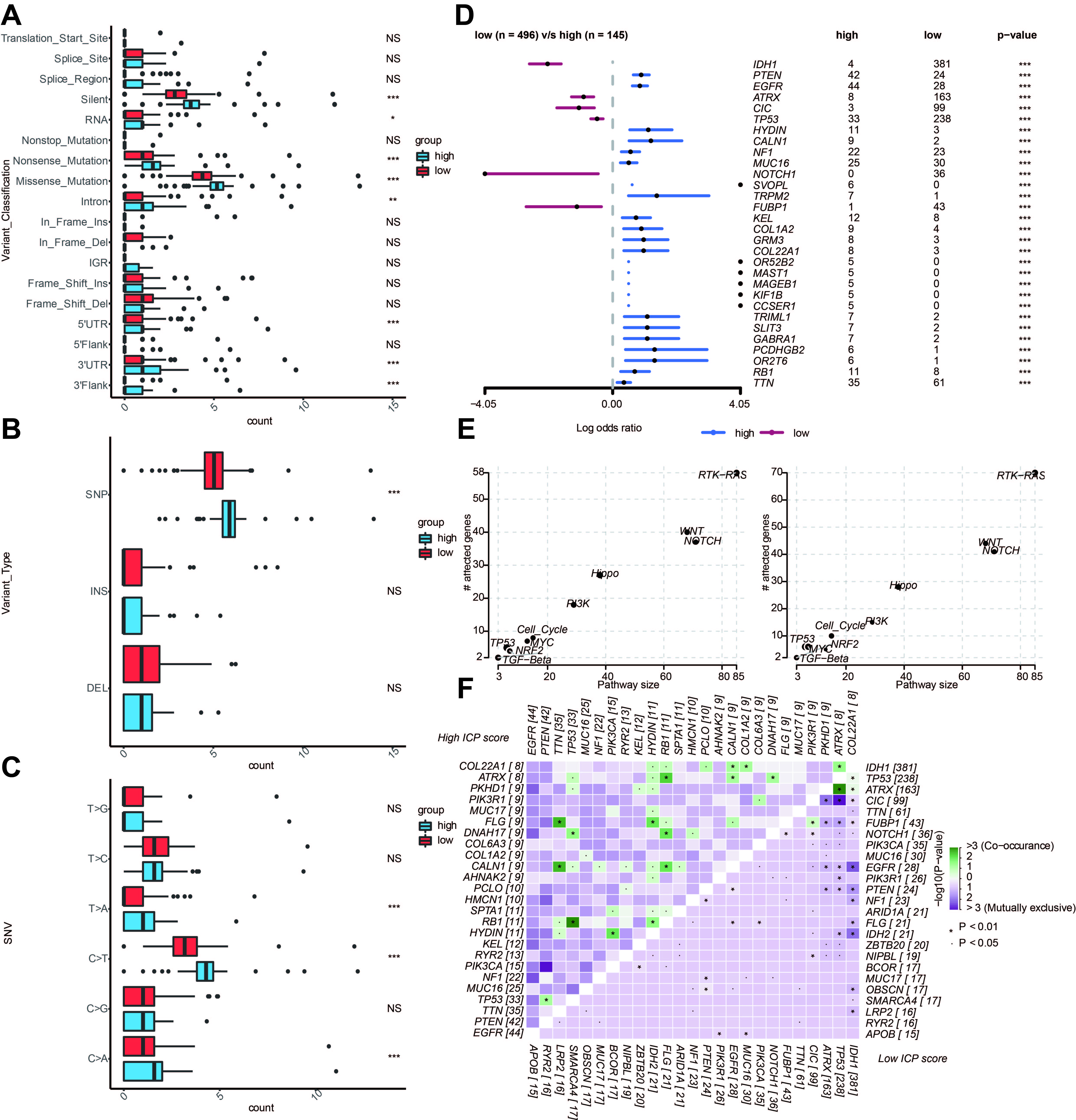


Figure S3. Box plot showing the mutation frequency comparison according to **A**. types of mutation, **B**. INDEL and SNP, and **C**. SNV between the two ICP score groups. **D**. Forest plot listing the top 30 most mutated genes between the two ICP score groups. **E**. Pathways involved in cancer biology in the two ICP score groups. **F**. Heatmap depicting the Co-occurance or Mutual exclusivity of the top 25 most mutated genes in the two ICP score groups.


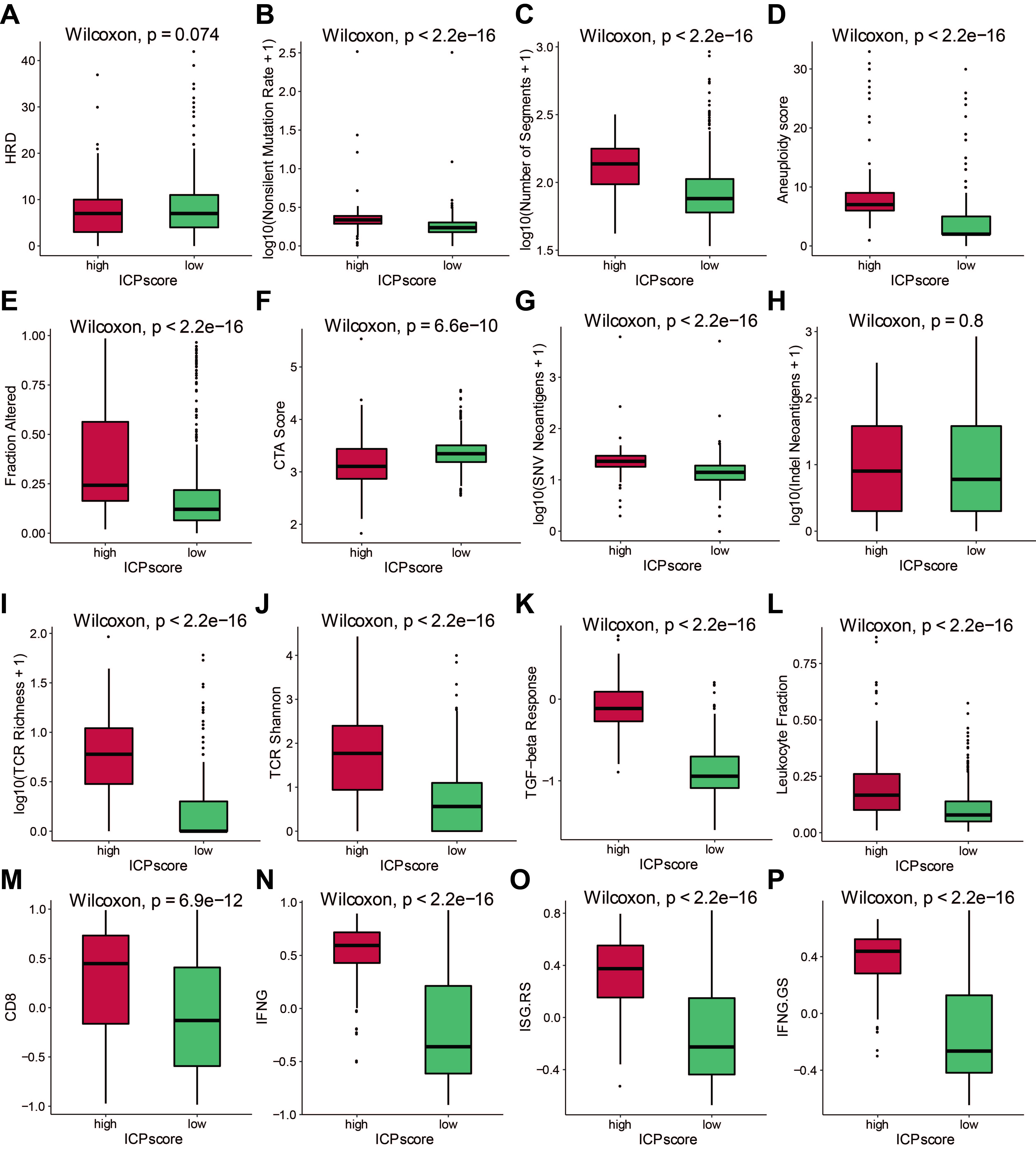


Figure S4. Assessment of tumor immunogenicity and antigen presentation capacity in the two ICP score groups. Comparison of 16 indicators (including HRD (**A**), nonsilent mutation rate (**B**), number of segments (**C**), aneuploidy score (**D**), fraction altered (**E**), CTA score (**F**), SNV neoantigens (**G**), Indel neoantigens (**H**), TCR Richness (**I**), TCR Shannon (**J**), TGF-beta Response (**K**), leukocyte fraction (**L**), CD8 (**M**), IFNG (**N**), ISG.RS (**O**), and IFNG.GS (**P**)) associated with tumor immunogenicity among two ICP score groups.


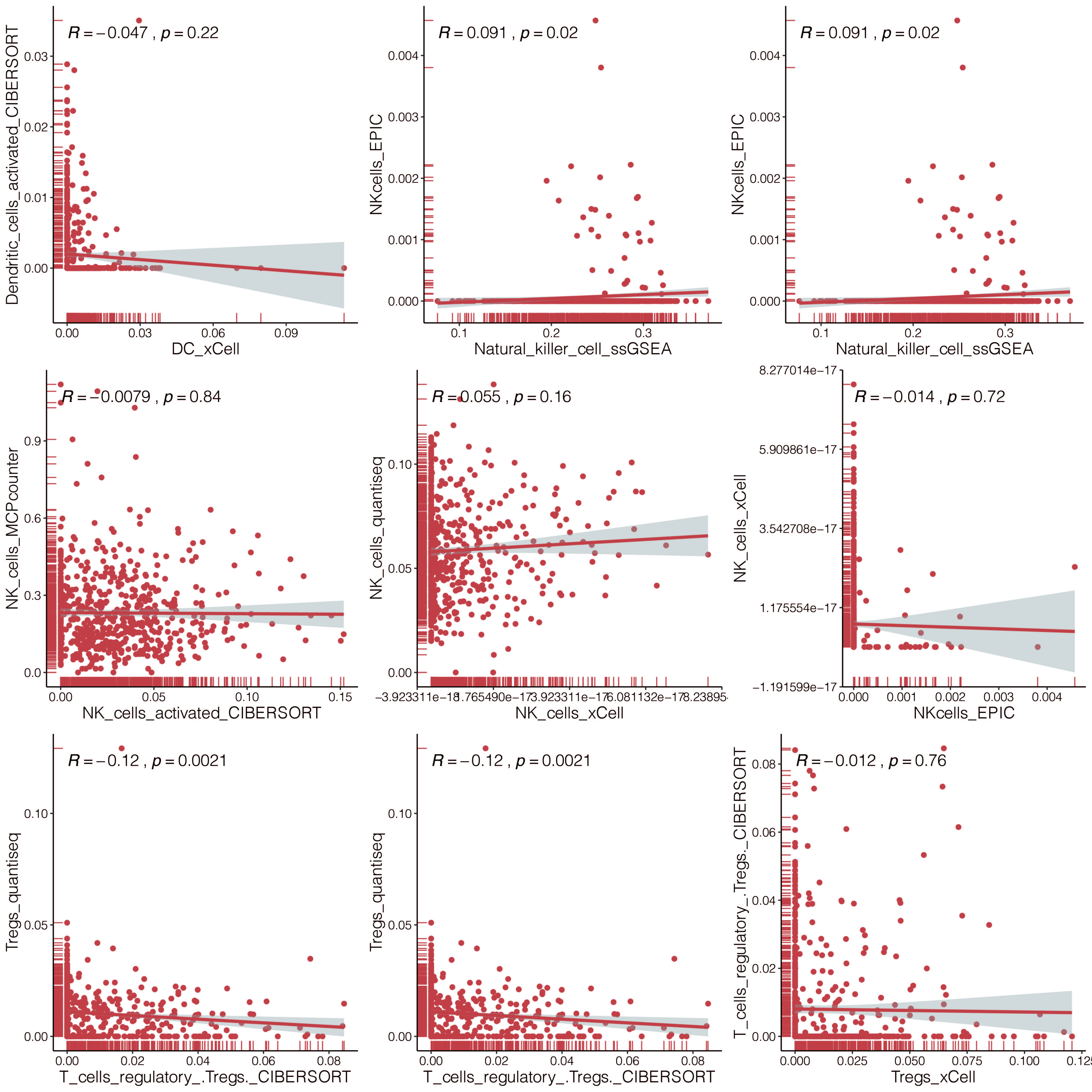


Figure S5. Correlation between immune cells calculated by different reference genome from different algorithms.

**Supplementary Tables**

Table S1. Basic information of fourteen included gliomas cohorts

Table S2. 65 immune cell types

Table S3. Univariate Cox analysis about immune cells in TCGA, CGGA, GSE108474 and the overlapped prognostic cells

Table S4. ICP score constructed in Xiangya cohort

Table S5. ICP score constructed in six independent cancer types in TCGA

Table S6. Significantly different CNV regions compared between two ICP score groups in TCGA

Table S7. Indicators for the assessment of immunogenicity and antigen presentation capacity

Table S8. ICP score constructed in IMvigor210

Table S9. ICP score constructed in GSE78220
